# Prevalence of Human Sapovirus in Low and Middle Income Countries

**DOI:** 10.1155/2018/5986549

**Published:** 2018-09-02

**Authors:** Mpho Magwalivha, Jean-Pierre Kabue, Afsatou Ndama Traore, Natasha Potgieter

**Affiliations:** ^1^Department of Microbiology, School of Mathematical and Natural Sciences, University of Venda, South Africa; ^2^Dean of School of Mathematical and Natural Sciences, University of Venda, South Africa

## Abstract

**Background:**

Sapovirus (SV) infection is a public health concern which plays an important role in the burden of diarrhoeal diseases, causing acute gastroenteritis in people of all ages in both outbreaks and sporadic cases worldwide.

**Objective/Study Design:**

The purpose of this report is to summarise the available data on the detection of human SV in low and middle income countries. A systematic search on PubMed and ScienceDirect database for SV studies published between 2004 and 2017 in low and middle income countries was done. Studies of SV in stool and water samples were part of the inclusion criteria.

**Results:**

From 19 low and middle income countries, 45 published studies were identified. The prevalence rate for SV was 6.5%. A significant difference (*P=0*) in SV prevalent rate was observed between low income and middle income countries. Thirty-three (78.6%) of the studies reported on children and 8 (19%) studies reported on all age groups with diarrhoea. The majority (66.7%) of studies reported on hospitalised patients with acute gastroenteritis. Sapovirus GI was shown as the dominant genogroup, followed by SV-GII.

**Conclusion:**

The detection of human SV in low and middle income countries is evident; however the reports on its prevalence are limited. There is therefore a need for systematic surveillance of the circulation of SV, and their role in diarrhoeal disease and outbreaks, especially in low and middle income countries.

## 1. Introduction

An estimated number of 6.3 million deaths of children under the age of 5 years suffering from diarrhoea have been reported worldwide [1, 2]. In Africa, death due to diarrhoeal disease remains a major health concern, though it has decreased from 2.6 million to 1.3 million between 1990 and 2013 [3]. Diarrhoeal disease is the important cause of morbidity and mortality in low and middle income countries, also the third most frequent cause of death and greatest contributor to the burden of disease in children younger than 5 years of age [4]. The infection of human intestinal tract occurs through transmission at the household level due to different pathways such as ingestion of contaminated food and water, poor waste disposal, and person-to-person interactions in the households and community [4, 5]. Low and middle income countries still face challenges like inadequate human waste disposal, poor water quality, poor health status, and disease transmission through faecal-oral route [6].

Amongst diarrhoeal causing agents, Sapovirus (SV) is one of the enteric viruses that cause acute gastroenteritis in humans and animals. Sapoviruses were previously called “typical human Caliciviruses” or “Sapporo-like viruses” in the family Caliciviridae [7]. They are identified as nonenveloped, positive-sense, single-stranded ribonucleic acid (RNA) genome of approximately 7.1 to 7.7 kb in size with a poly(A) tail at the 3'-end [8–10]. Amongst the five designated genogroups (GI to GV), GIII infects porcine species [11–14], while GI, GII, GIV, and GV infect humans [15]. Currently, human SV genogroups are classified into 16 genotypes (comprising seven genotypes for GI and GII, respectively, and one genotype each for GIV and GV) through phylogenetic analysis of the complete capsid gene [15, 16]. Coinfections of SVs with other enteric viruses (such as noroviruses [NoVs], rotaviruses [RVs], astroviruses [AstVs], adenoviruses [AdVs], enteroviruses [EVs], and kobuviruses [KbVs]) have been noted in acute gastroenteritis outbreaks in humans [17–19].

This review summarises reports on SV detection and typing in low and middle income countries. In addition, it highlights the need to establish the relatedness of circulating SV strains in environmental (water) samples and clinical samples from communities in low and middle income countries (particularly rural settings). The time-frame chosen was 2004 to 2017 because of the availability of published data on human SV within the low and middle income countries.

## 2. Methodology

Two literature searches were carried out. The first literature search was performed using the terms: calicivirus, sapovirus, and developing countries, as listed by National Institutes of Health PUBMED library and ScienceDirect. A second literature search was independently done for each of the 139 “developing” countries accessed from the list published by the Society for the Study of Reproduction (http://www.ssr.org). Furthermore, the identified countries were then assessed according to the 2018 World Bank analytical classification report (http://datahelpdesk.worldbank.org/knowledgebase/articles/906519). For a successful search, each of the countries' names was combined with the following keywords: calicivirus, sapovirus, enteric viruses, and gastroenteritis. Studies identified by the search terms were selected for inclusion in the review based on the following inclusion criteria:

- Studies limited to human SV detected in clinical specimen and environmental water samples, reported in the 21st century.
- SV studies using laboratory molecular techniques including nested-PCR (nPCR), real time-PCR (RT-PCR), and RT-multiplex PCR.

Studies were excluded from the review if SV was detected in other mammalian species or animals or if the study was conducted in high income countries. In case of duplication of studies by authors, only one article was included.

Data was extracted from each selected study when provided: country name and its economic status (i.e., low income, lower, and upper middle income) as per the analytical classification report by World Bank, study setting (hospitalised, outpatient, and environment), study population (age group), population size, duration of the study, diagnostic method used, number of samples tested for SV (including their genogroups and genotypes), first author, and year of publication (Tables 1, 2 and 3).

The difference of SV data in middle and low income countries was analysed for statistical significance by Student's t-test using the simple interactive statistical analysis (SISA) at http:home.clara.net/sisa. Result with* P* < 0.05 was considered significant.

## 3. Results

**Figure 1:**
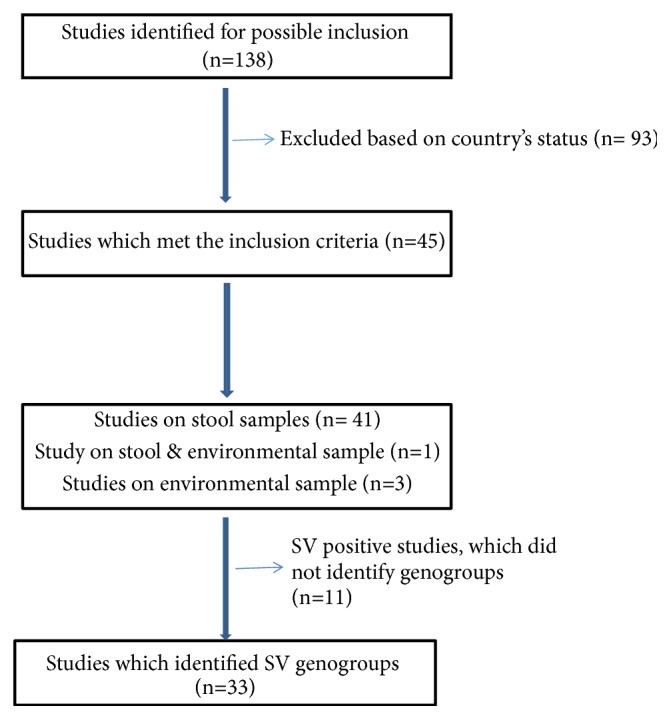
Schematic diagram showing search process for selection of studies reported.

A total of 138 articles published from 2004 to 2017 were identified from 19 low and middle income countries. After selection based on the selection criteria (Figure 1), a total of 45 studies met the inclusion criteria. From 45 publications, 41 reported on clinical (stool) samples, 3 on environmental (water) samples, and 1 on both. Of the 42 studies conducted on clinical specimens, 66.7% (n=28) were done in hospitalised patients, 23.8% (n=10) in outpatients, and 9.5% (n=4) in both hospitalised and outpatient settings.

### 3.1. SV Age Distribution in Human Populations

The majority of studies (78.6%; 33/42) investigated SV in children less than 5 years of age and a further 19% (8/42) included all ages. However, only a single study investigated SV in adults with diarrhoea or acute gastroenteritis.

### 3.2. Seasonality

The detection of SV from clinical samples based on seasonality was reported in only 14.3% (6/42) of the studies. The majority (42.9%, 18/42) of the studies did not report on the time-frame of detection, 38% (16/42) of the studies showed inconsistent time-frame of detection, and 4.8% (2/42) of the studies showed detection throughout the year. Studies investigating SV in water sources in South Africa (SA) did not detect any seasonal peaks.

Five studies reported on samples collected within a period of 2 to 4 months, and these cases were not defined as outbreaks, while the duration period of sample collection for other 40 studies ranged over periods from 1 year to 5 years.

### 3.3. Sapovirus Detection and Genotyping

From the 42 included studies, 41 of these reported SV positive cases while only one study on adults reported negative results (Tables 1 and 2). Mixed infection of SV with bacteria and/or other enteric viruses was identified in 19.5% (8/41) of the studies, a SV single strain was identified in 36.6% (15/41) of the studies, and mixed strains of SV were identified in 43.9% (18/41) of the studies. From the 41 studies, only 31 studies reported SV detection with identification of the genogroups/genotypes. Overall detection of SV strains showed SV-GI.1 and GI.2 as the most dominant [90%  (28/31)] strain from different settings of studies, followed by SV-GII.1, GII.2, GII.3, and GII.4 with the least detection of SV-GIV strain and –GV (GV.2) strain. No study showed the occurrence of SV-GIV as a single detection but only in mixed infection cases.

The prevalence rate of SV from the 41 documented studies in low and middle countries was 6.19% with a range from 0.2% to 39%. Further breakdown showed significant difference (*P *=0) in SV prevalence rate between low income (10.40%) and middle income (5.86%) countries. Although data on the prevalence of SV in African countries is limited, thus far, eight studies have been conducted in urban settings. Detection of SV from children in Africa is recorded with different incidence rates: in Tunisia [0.8%] [57], Burkina Faso [18%, 10.3%, respectively] [16, 51], and South Africa [4.1%, 7.7%, respectively] [53, 56]. The prevalence of SV in all ages was reported from South Africa [8.4%] [54], Ethiopia [4.2%] [52], and Kenya [4%] [3]. A predominance of SV-GIV (53/221, 24%) was noted in the South African study done on stool samples from hospitalised children with gastroenteritis [55].

Only 8.9% of studies reported SV in the environmental and waste water samples from low and middle income countries. The detection of SV-GI, SV-GII, and SV-GIV has been reported from polluted water sources by wastewaters and also on samples collected from treatment plants within selected areas of SA [58–60]. Sapovirus genogroups I and II were identified from river water samples, with detection rate of 48.5% (48/99) [58], while, in Brazil, SV-GI (genotypes 1 and 2) were detected (33%, 51/156) from the wastewaters [22], Table 3.

## 4. Discussion

This review provides a summary of studies conducted in developing countries on the detection of human SV. Only 45 (41 stool samples, 3 water samples, and 1 both stool and water sample) studies satisfied the inclusion criteria of this review highlighting the importance for systematic surveillance monitoring human SV circulating in developing countries (rural and urban communities). Very little is known about the contribution of human SV to diarrhoeal disease in developing countries; this is reflected in the fact that reported studies were only from 19 identified countries which include 5 African countries, namely, Burkina Faso, Ethiopia, Kenya, South Africa, and Tunisia (Table 2). A total of 78.6% (33/42) studies reported on children ≤5 years of age from the collected data, highlighting the role of SV in diarrhoeal disease amongst children in the developing countries. Hence, SV and other emerging enteric viruses, being underappreciated, can be an important cause of Norovirus negative outbreaks as reported by Lee and colleagues [61]. In addition, since it is difficult to culture human SV on cell lines [13], specialised molecular laboratories are needed for the investigation of such virus in the developing countries. Because of lack of funding and skills, the prevalence of enteric viruses is underreported in Africa and other developing countries [62]

Most of the studies (66.7%; 28/42) were done in hospitalised patients, and this might be due to the fact that SV infection sometimes leads to hospitalisation as illustrated from other studies [49, 63]. GEMS study reported SV amongst other enteric pathogens to have been associated with moderate to severe diarrhoea in developing countries [64]. The Millennium Development Goals (MDG) 2015 report shows disadvantaged settings being vulnerable as compared with the advantaged or developed settings, highlighting the effectiveness and affordability of treatments, and improved service delivery and political commitment playing a role in such settings. The statistical analysis of this review similarly showed a significant difference in the prevalence of SV in low income than in middle income countries (*P*=0).

The circulation of SV genogroups shows variability, with SV-GI and SV-GII detected frequently, while SV-GIV and SV-GV are rarely detected comparing to other genogroups [16]. An African study (Burkina Faso) reported SV-GII as the predominated strain, mostly in outpatients with diarrhoea (81.5%: 22/27), suggesting that this genogroup may be less virulent and require fewer hospital admissions. However, additional studies on outpatients will have to be conducted to confirm this observation. Although the detection of SV-GII is seen in diarrhoeal samples, it might be less virulent to cause severe symptoms leading to hospitalisation of patients, unlike SV-GI which is commonly known to be associated with severe symptoms and frequently detected in patients presenting with gastroenteritis [16, 32]. The detection of SV (GI, GII, GIV, and GV) in gastroenteritis outbreak cases has been reported in high income countries, however with less detection rate of SV-GII in both cases [14, 17, 61, 65].

Human SV infections cases relating to acute gastroenteritis in people of all ages have been identified worldwide [14]. Notwithstanding the potential selection biases present based on the studies available for inclusion, this review shows that the prevalence in children may be higher than in adults in low and middle income countries. In addition, the GEMS study in low and middle income countries highlights diarrheal disease in children as a leading cause of illness and death and also increasing the risk of delayed physical and intellectual development [66]. It has been reported that sporadic and outbreak cases caused by enteric viruses spread mainly by person-to-person contact, contaminated surfaces or objects, and contaminated water or food [67]. Therefore children are more vulnerable than adults within such exposed environment, probably because of immune system development. However, previous studies noted that gastroenteritis symptoms are usually self-limiting, and patients usually recover within a couple of days depending on the individual immune's response [49, 63]. Adults are likely to consider self-treatment by oral rehydration solution (ORS) which is the safe, effective, and low cost therapeutic option preventing dehydration [68], hence not consulting in healthcare facilities or likely due to self-respect.

Sapoviruses, like other enteric viruses, play an important role in the burden of disease worldwide. The GEMS conducted a three-year study in selected low and middle income countries, amongst children aged 0 to 59 months, and reported the detection of SV (3.5%) associated with diarrhoea [64]. However, there is no surveillance system on SV infection and prevalence in low and middle income countries, which means underreporting of sporadic cases of human SV and its epidemic are underestimated. Nevertheless, detection and comparison of the SV strains circulating in low and middle income countries (especially Africa) are currently underreported and this could be due to various techniques used for sampling and detection, including study site conditions.

Information on seasonality, patient history, area settings, and predicated pattern of transmission of viruses within the community provides knowledge needed to implement public health intervention strategies. Furthermore, detection of enteric viruses (such as SV) in environmental samples gives awareness of the circulation of infectious viral particles within the population and health-hazards which might be associated with the environment. The predictable effects of human waste disposal, water quality, and high rate of immunocompromised society have been a big concern in low and middle income countries, but there are still few documented reports on the detection of SV from environmental samples. This is highlighted by the finding of this study with high prevalence of SV in low income countries. The survival and development of children depend on good hygiene practices and use of clean drinking and domestic water on daily basis [4]. Monitoring of genetic diversity of the current circulating or emerging SV genogroups, possible water-borne transmission, and possible zoonotic infections amongst the communities is critical, and studies which can show the transmission of SV between the environment(s) (especially river water), domestic animals, and human should be considered, and the role that SV plays in diarrhoeal diseases [69].

## 5. Conclusion

This review found substantial evidence of SV proportion associated with diarrhoeal disease in low and middle income countries. However there is limited data reporting the detection of circulating SV strains. Therefore systematic surveillance of SV circulation within the communities in low and middle income countries is needed to assess sufficiently its role in diarrhoea disease.

## Figures and Tables

**Table 1:** Summary of human SV detection from 33 studies (stool samples) conducted in 14 non-African low and middle income countries.

**Country**	**World Bank Classification as of year 2018**	**Study setup**				**Prevalence (seasons or defined period of incidence)**	**Method used**	**Rate of Detected Genotypes**	**Reference**
		**Study population**	**Population size**	**Study setting**	**Duration of study**				
***Bangladesh***	Lower middle income	Infants/ Children	917	HP with AGE	From 2004 to 2005	Oct 2004 – Jan 2005, Sept 2005	RT-PCR	2.7 % SV (***All in <3 yrs of age***) SV-GI.1, GI.2	Dey et al [20]

***Brazil***	Upper middle income	Children	305	HP severe GE	From March to September 2003	March, May - September	RT-PCR	15/305 (4.9%), mixed infection of SV and Astv in 1 sample SV-GII.1, SV-GI.1, SV-GI.2	Aragao et al [21]
		Children (0 – 10 yrs	159	OP (81 = diar; 78 = non-diar)	From April 2008 to July 2010	February, April	RT-PCR	2 of 81: 2.5% SV (GI.1, GII.2)	Aragao et al [22]
		Children (6-55 mn old)	539	Day Care (Healthy)	From October 2009 to October 2011	Not defined	RT- multiplex PCR	25/539 (4.6%) SV, SV-GI.1, GI.3	de Oliveira et al [23]
		Children, outpatients	212 129	HP OP With AGE	From 2012 to 2014	Not defined	Quantitative real-time PCR (qPCR)	12/341 (3.5%) **[9/12 – HP, 3/12 – OP].** SV-GI.1 dominant, GI.2, GI.6, GII.1, GV.1	Fioretti et al [24]
		Children < 10yrs	426 (156 of <3yrs tested)	HP with AGE	From January 2010 to October 2011	Aug & Sept	RT-PCR	6/156 (3.8%), SV-GI.1, GI.2, GII.2, GII.4	Reymao et al [25]
		Children	172	Community	From 1990 t0 1992	Not defined	Nested PCR	9/172 (5.2%) SV-GI.1, GI.7, GII.1, GV.2	Costa et al [26]

***China***	Upper Middle income	Children <5yrs old	500	OP with acute (477)/ persistent (23) diar	From August to November 2010	Aug – Nov 2010	RT-PCR	9/477: 1.89% SV ***(<24 month children***), mixed infection of SV & AdV in 1 sample, SV-GI dominant, SV-GII & SV-GIV	Ren et al [27]
		Patients (1mn – 78yrs)	412	HP & OP with AGE	From August 2014 to September 2015	Not defined	RT-PCR	[9/412] 2.2% SV single infection, Co-infection: 2/412 ETEC with SV, 1/412 Salmonella sp with SV, 1/412 Salmonella sp with SV & AdV **Genogroups not defined**	Shen et al [28]

***India (New Delhi)***	Lower middle income	Children <10yrs	226	HP with AGE	From August 2000 to December 2001	Not defined	Multiplex two-step RT-PCR	23/226 (39%), mixed infection in 5 samples {NV-GII and SV-GI} SV-GI [22], GII [1]	Rachakonda et al [29]

***Iran***	Upper middle income	Children	200	HP with AGE	From 2008 to 2009	Winter and in fall	RT-PCR	6/200 (3%), SV-GII	Parsa-Nahad et al [30]
		Patients (3 mn - 69yrs; mean 15.3yrs	42	HP with AGE	From May to July 2009	May – July 2009	RT-PCR	11.9% SV (***patients with <5yrs of age***) SV-GI.2	Romani et al [31]

***Mongolia***	Lower middle income	Infants	36	households	From July to August 2003	Jul – Aug 2003	RT-PCR	1/36 (2.8%) pos for SV SV-GI	Hansman et al [11, 12]

***Nicaragua***	Lower middle income	Children <5yrs	330	(175 HP; 155 OP), with AGE /diar	From September 2009 to October 2010	Nov 2009- Feb/Mar 2010, May-Aug/Sept 2010	Real-time PCR	57/330 (17%): **HP = 15% [27/175], OP = 19% [30/155]**. SV-GI, GII, GIV {HP: GI.1, GI.2; OP: GII.2, GII.3	Bucardo et al [32]

***Pakistan***	Lower middle income	Infants <6 to >35 mn	122 Pos: Enteric Viruses	HP with AGE	From 1990 to 1994	Mar, Aug - Oct	RT-PCR	13.9% SV detection (12.3% SV mono-infections, 1.6 mixed infection – AstV & SV), SV-GI	Phan et al [33]
		Infants & children <1 mn – 5yrs	517	HP with AGE	From 1990 to 1994	1990: Aug, Sept, Oct 1991: Jan, May, Jul, Oct 1992: Mar, Aug, Sep 1993: Sep 1994: Apr, July	RT-PCR	3.2 % SV SV-GI dominated, followed by GII, and GIV	Phan et al [34]

***Papua New Guinea (Goroka)***	Lower middle income	Children <5yrs	199	HP with AGE	From August 2009 to November 2010	Not defined	RT-PCR	4/199 (2%) SV, **Genogroups not defined**	Soli et al [35]

***Peru***	Upper middle income	Children <2yrs	599	300 non-diar, 299 diar	From 2007 to 2010	Four seasons	Quantitative reverse transcription-real-time PCR (qPCR)	9.0% overall: *∗ ***12.4**%** [37/299] diarrhoeal **– SV-GI/1/2/6/7, GII.1/2/4/5, GIV, GV/1; *∗ ***5.7**%** [17/300] non-diarrhoeal** – SV-GII.5, GIV	Liu et al [36]

***Philippines***	Lower middle income	Children <5yrs	417	HP with AGE	From June 2012 to August 2013	Not defined	Real-time PCR	29/417 (7%) detection, (co-infection in 10/29: 6/10 with RV, 2/10 with NV, 2/10 with AstV). SV-GI.1, GI.2, GII.1, GII.4 & GV	Liu et al [1, 2]

***Thailand***	Upper middle income	Infants	80 randomly selected	HP with AGE	From November 2002 to April 2003	Nov 2002 – April 2003	RT-PCR	15%: 11% single infection, 4% mixed infection – NoV & SV), SV-GI	Guntapong et al [37]
		Children <5yrs	248	HP with AGE	From 2002 to 2004	Not defined	RT-PCR	3/248 (1.2%) SV- single infections SV-GI [GI.1 &GI.2], GIV	Khamrin et al [38]
		Children	296	HP with AGE	From May 2000 to March 2002	Jun-Jul, Jan-Mar, May-Jul, Mar.	RT-PCR	25%, mixed infection I 1 sample (NV-GI and SV) SV-GI.1, GI.4, GI.5, GII.1, GII.2	Malasao et al [39]
		All age groups	273	HP with AGE/diar	From January 2006 to February 2007	Early summer: March & April	RT-PCR	0.8% SV SV-GII/3	Kittigul et al [40]
		Children (Neonate to 5yrs old)	147	HP with AGE/watery	January to December 2005	Not defined	RT-PCR	5/147 (3.4%) SV SV-GI [GI.2, GI.1, GI.5] dominating, SV-GII.3	Khamrin et al [41]
		Pediatric patients	160	HP with AGE	January to December 2007	Throughout the year	RT-multiplex PCR	5/160 (3.1%) SV **Genogroup not defined**	Chaimongkol et al [42]
		Children <5yrs	567	HP with AGE	In 2007, and from 2010 to 2011	2007: Feb, Sept, Oct. & 2010: Dec	Semi-nested RT-PCR	7/567 (1.2%), SV-GI.1	Chaimongkol et al [43]
		Adult (15yrs – 90yrs)	332	HP with diar	Year 2008	Not defined	RT- multiplex PCR	***No SV detected***	Saikruang et al [44]
		Patients	1141	HP with AGE	From 2006 to 2008	May - July	RT-PCR	1.1% SV, mixed infection of NoV-GII & SV in 2 samples **Genogroup not defined**	Pongsuwanna et al [45]

***Vietnam***	Lower middle income	Children	448	HP with acute sporadic gastroenteritis	From December 1999 to November 2000	Not defined	RT-PCR	1/448 (0.2%) SV SV-GI	Hansman et al [46]
		Paediatric patients	1010	HP with viral AGE	From October 2002 to September 2003	Oct 2002 – Sep 2003, Rainy season (July)	RT-PCR	0.8% SV (0.4% monoinfection, 0.4% coinfection), **Genogroup not defined**	Nguyen et al [47]
		Pediatric	502	HP with AGE	From December 2005 to November 2006	Dry season	RT-PCR	1.2% SV	Nguyen et al [48]
		Children <5yrs	501	HP with AGE	From November 2007 to October 2008	Cooler months (Oct – Feb)	Real-time RT-PCR	1.4% SV SV-GI and SV-GII Co-infection of (NoV & SV) in 1 sample, of (NoV, SV, and RV) in 1 sample	Trang et al [49]

*Independent States of the former * *** Soviet Union***	See information below describing the States	Children	495	HP with AGE	From January to December 2009	Jan - Mar, May – Aug	Real-time PCR	16/495 (3.2%) SV-GI.1 dominating	Chhabra et al [50]

HP = hospitalised patient; OP = outpatient; AGE = acute gastroenteritis; mn= month; yr(s) = year(s); diar = diarrhoea; SV = Sapovirus; G (I-IV) = genogroup (I-IV)

**∗** ***Independent States of the former Soviet Union*** *refers to* ***Armenia, Azerbaijan & Belarus (*** *upper middle income status* ***), and Georgia, Republic of Moldova & Ukraine (*** *lower middle income status* ***).***

**Table 2:** Summary of human SV detection from 9 studies (stool samples) conducted in 5 African countries.

**Country**	**World Bank Classification as of year 2018**	**Study setup**				**Prevalence (seasons or defined period of incidence)**	**Method used**	**Rate of Detected Genotypes**	**Reference**
		**Study population**	**Population size**	**Study setting**	**Duration of study**				
***Burkina Faso***	Low income	Children	263 diarrhoeal, 50 non-diarrhoeal	Urban area (HP & OP)	From November 2011 to September 2012	Not defined	Real-time RT-PCR	9%: 27/263 (10.3%) {***5/27 = hospitalised, 22/27 = non-hospitalised***} & 3/50 (6%) SV-GII [GII.2, GII.1, GII.3], SV-GI.2	Ouedraogo et al [51]
		Children <5yrs	309 diarrhoeal	Not defined	From May 2009 to March 2010	Not defined	Real-time PCR	56/309 (18%) [mixed infection: with RV 25/56, with NV 5/56; single infection 20/56] Genogrouping {34/56}: SV-GI [GI.1, GI.4], GII [GII.1, GII.4, GII.6], GIV.1 & GV.1	Matussek et al [16]

***Ethiopia***	Low income	All age groups	213 diarrheic samples	Government Health Care Centre	From June to September 2013	June-sept 2013	RT-PCR	9/213 (4.2%) One sequenced (SV-GII.1)	Sisay et al [52]

***Kenya***	Lower middle income	All age groups	334-Lwak & 524-Kibera.	Clinics with diar	From June 2007 to October 2008	Not defined	RT-PCR	5%: 13/334 (4%) and 31/524 (6%) SV **Genogroups not defined**	Shioda et al [3]

***South Africa***	Upper middle income	Paediatric <13yrs	245	HP gastroenteritis	Year 2008	Not defined	Real-time RT-PCR	10/245 (4.1%) incl. one Mixed infection with NV **Genogroups not defined**	Mans et al [53]
		Patients 1mn to 87yrs mean 14yrs	190 94 diar 93 non-diar 3 unknown	Bio-wipes from rural households	From July 2007 to December 2008	Not defined	Real-time RT-PCR	16/190 (8.4%): (1 - 62yrs: mean 24yrs) **Genogroups not defined**	Mans et al [54]
		Children	Selected) 296 of 477 SV-Pos (for characterisation)	HP with gastroenteritis	From April 2009 to December 2013	Not defined	Nested PCR	221 were characterised (genotyped) SV-GI [GI.1 – GI.3, GI.5, GI.6, GI.7], SV-GII [GII.1 – GII.7], SV-GIV	Murray et al [55]
		Children <5yrs	3103	HP diar	From 2009 to 2013	Higher in Summer & Autumn (Nov to Apr)	Real-time PCR	238/3103 (7.7%) SV **Genogroups not defined**	Page et al [56]

***Tunisia***	Lower middle income	Children	788 [408 HP, 380 OP]	Consulting for AGE	From January 2003 to April 2007	Not defined	RT-PCR Primer Noel, 1997	6/788 (0.8%) [Mixed infection: with RV 2/6; single infection 4/6]. Positive from OP samples SV-GI.1	Sdiri-Loulizi et al [57]

HP = hospitalised patient; OP = outpatient; AGE = acute gastroenteritis; mn= month; yr(s) = year(s); diar = diarrhoea; SV = Sapovirus; G (I-IV) = genogroup (I-IV).

**Table 3:** Summary of human SV detection from 4 studies (water samples) conducted in low and middle income countries.

**Country**	**World Bank Classification as of year 2018**	**Samples**			**Prevalence (season)**	**Method used**	**Rate of detection**	**Reference**
		**Type**	**Size**	**Duration**				
Brazil	Upper middle income	Wastewater	156	From 2012 to 2014	Summer and Autumn	Quantitative real-time PCR (qPCR)	51/156 (33%)	Fioretti et al [24]

South Africa	Upper middle income	River water	99	From 2009 to 2010	May, Aug, Nov (2009); Jan, April (2010)	RT-PCR	48/99 (48.5%)	Murray et al [58]
		Wastewater	51	From August 2010 to December 2011	August (2010), June, July (2011)	Real-Time qPCR	37/51 (72.5%)	Murray et al [59]
		Water (various source)	10	January and March 2012	January and March 2012	Real-Time PCR	8/10 (80%)	Murray and Taylor [60]
